# UNIFIED: Understanding New Information from Emergency Departments Involved in the San Bernardino Terrorist Attack

**DOI:** 10.5811/westjem.2019.11.43437

**Published:** 2020-02-24

**Authors:** Dustin Smith, Elizabeth L. Walters, Ellen Reibling, Darren Brockie, Carol Lee, Michael Neeki, Humberto Ochoa, Travis Henson, James Fisgus, Tammi Thomas

**Affiliations:** * Loma Linda University Health, Department of Emergency Medicine, Loma Linda, California; † Arrowhead Regional Medical Center, Department of Emergency Medicine, Colton, California; ‡ Riverside University Health System, Department of Emergency Medicine, Moreno Valley, California; § St. Bernardine’s Medical Center, Department of Emergency Medicine, San Bernardino, California; ¶ San Antonio Regional Hospital, Department of Emergency Medicine, Upland, California

## Abstract

**Introduction:**

Emergency departments (ED) are on the front line for treating victims of multi-casualty incidents. The primary objective of this study was to gather and detail the common experiences from those hospital-based health professionals directly involved in the response to the San Bernardino terrorism attack on December 2, 2015. Secondary objectives included gathering information on experiences participants found were best practices.

**Methods:**

We undertook a qualitative study using Consolidated Criteria for Reporting Qualitative Research (COREQ) guidelines by performing semi-structured interviews with physicians, nurses, and incident management staff from multiple institutions responding to the San Bernardino terrorist attack. We coded transcripts using qualitative analysis techniques and we delineated and agreed upon a refined list with code definitions using a negotiated group process. Final themes were developed and analyzed.

**Results:**

A total of 26 interviews were completed; 1172 excerpts were coded and categorized into 66 initial themes. Six final categories of communication, training, unexpected help, process bypassed, personal impact/emotions, and practical advice resulted.

**Conclusion:**

Our study provides context regarding the response of healthcare personnel from multiple institutions to a singular terrorist attack in the United States. It elucidates several themes to help other institutions prepare for similar events. Understanding these common experiences provides opportunity to prepare for future incidents and develop questions to study in future events.

## INTRODUCTION

Active shooter incidents, a subcategory of mass casualty incidents (MCI), while relatively rare, are increasing in the United States (US). The average of 11.4 active shooter incidents between 2000–2013 increased to 20 each year 2014–2016, and 30 in 2017.^1^,^2^ Casualty numbers during 2016–2017 were higher than prior years due to the incidents at the Route 91 Harvest Festival in Las Vegas, NV, Pulse Nightclub in Orlando, FL, and the First Baptist Church in Sutherland Springs, TX.^3^ MCIs are “an imbalance between the numbers of injured who need medical care and the medical ability of emergency systems to deliver optimal care to each individual.”^4^ In the 2013 Boston Marathon bombing, 118 people were transported to nine hospitals in 18 minutes with more than 264 seeking treatment.^5^ During the response to the Route 91 Harvest Festival mass shooting incident in 2017, the University Medical Center of Southern Nevada cared for 104 patients, Sunrise Hospital and Medical Center received 212 patients, and St. Rose Dominican Hospital cared for 37 patients.^6^ Hospitals must prepare for MCIs.^4^,^7^–^10^

On December 2, 2015, a terrorist attack in San Bernardino killed 14 and injured 22. Incident details were described in an earlier publication.^11^ The response involved six local hospitals in a regional network, the Inland Counties Emergency Medical Agency, 12 using ReddiNet^13^ (ReddiNet, Los Angeles CA), a communications network (Table 1). Previous studies have illuminated hospital responses to terrorist attacks.^14^–^16^ Common experiences of individual health professionals responding to a singular event are less well understood.

### Importance

Understanding common experiences of health professionals from different medical centers responding to the same, singular terrorist attack may provide new insights into shared challenges, best practices, and lead to questions worthy of additional study. Our study is the largest qualitative study of healthcare professionals responding to terrorism in the US. Previous studies have focused on attacks in Europe or Israel, or on responders other than physicians and nurses (i.e., social workers).^17^,^18^

### Goals of This Investigation

Our primary objective was to gather and detail the common experiences from those hospital-based health professionals directly involved in the response to the San Bernardino terrorism attack. Secondary objectives included gathering information on experiences participants found were best practices. The analysis of this information should allow professionals to generate questions for further study as well as review and improve their current MCI planning.

**Population Health Research Capsule**

What do we already know about this issue?

Mass casualty incident (MCI) responses push the limits of individual hospital based providers. Institutional preparation is essential as incident numbers increase.

What was the research question?

What common experiences of hospital providers directly involved in a terrorist response inform improvements in MCI planning?

What was the major finding of the study?

Common Experience: communication, training, unexpected aid, process bypass, personal impact, practical advice.

How does this improve population health?

Insights inform improvements in MCI planning at both individual & institution level. Planning for after event processing is essential to support clinical providers.

## METHODS

### Study Design and Setting

We undertook a qualitative study using Consolidated Criteria for Reporting Qualitative Research (COREQ) guidelines by performing semi-structured interviews with physicians, nurses, and incident management staff from multiple institutions responding to the San Bernardino **t**errorist attack.^19^–^21^ We chose this approach because terrorist attacks on US civilian targets are relatively rare and the inductive approach uncovers a deeper understanding of elusive or unexpected responses to clinical problems by allowing for probing questions.^22^,^23^ The Loma Linda University Institutional Review Board approved the study as exempt. Participants provided verbal consent.

### Selection of Participants and Data Collection and Processing

Three emergency physicians and one public health PhD, trained in qualitative approaches, interviewed participants. We used purposive and then snowball sampling to select participants whereby we contacted facility medical directors and asked them to provide a list of potential interviewees. We recruited from multiple distinct hospitals to achieve data source triangulation, building a comprehensive and thorough model by using diverse data sources by recruiting.^23^ Interviews were conducted at participant’s hospital or by phone, per interviewee’s choice, January 13, 2016 – March 17, 2016, using a standardized interview guide (Table 2). All interviews were audiotaped and then transcribed by paid transcriptionists. Interviews lasted between 13–60 minutes with a median of 31 minutes. We interviewed until theoretical saturation was achieved as no new relevant ideas were mentioned by additional participants. Another indication of saturation was repetitive themes that supported the resulting model. 22, 24 We analyzed data using DEDOOSE version 7.5.16 (Los Angeles, CA).

### Primary Data Analysis

Researchers independently coded four transcripts using qualitative analysis techniques.^22^ We refined the list using a negotiated group process until code definitions were delineated and agreed upon. We developed final themes using an inductive process over multiple meetings. Once themes were identified, team members performed additional data analysis together to identify relevant contrarian viewpoints found within the themes. Study participants did not provide feedback, but we included de-identified quotations emblematic of the discovered themes.

## RESULTS

### Characteristics of Study Subjects

We completed 26 interviews with hospital-based responders (Table 3). One hospital system treating only one patient declined participation. Interview coding produced 1172 excerpts categorized into 66 initial themes, which collapsed to six general categories: communication, training, unexpected help, processes bypassed, personal impact/emotions, and practical advice.

### Main Results

#### Active shooter incidents challenge traditional communication channels

Active shooter incidents present challenges of scale and function by occurring unexpectedly, demanding resources that are not typically available on a regular day, and challenging pre-identified hierarchies and defined job descriptions. Study participants used multiple communication methods, including REDDINET, in- person conversations, handheld hospital “disaster phones” distributed for MCIs, two-way pagers, and work or personal cell phones for voice and texting. Additional resources included television, hospital computers, social media, and other phone apps to stream news reports. Despite using multiple communication methods, participants reported having an incomplete picture of what to expect.

Initially we got a lot more information, we were just trying to gather more information. The first call they said that it was 10–20 victims we didn’t know if they were coming to us, or how many or if all of them were coming to us.

I don’t know what’s coming in. I didn’t even know if it was a patient themselves or the shooter. It could have been either of them. No one knew anything.

Many respondents reported trusting information using peer-to-peer (PTP) methods such as text and Facebook messages. PTP methods seemed weighted more than other communication methods, especially when the messenger was a personal acquaintance. Physicians promptly responded to the PTP requests for resources, including residents at the trauma centers. It was an education day at two locations so many residents were on site. Administrative response was quick at all locations. For example, it was clear that administrators intervened to move patients up to floors to open ED beds for potential victims. On the other hand, the administrative response also increased ED traffic leading to potential confusion about who was in charge. The large number of available physicians created the potential for confusing communication.

For instance one of my colleagues was astute enough to recognize our communication difficulties; some of the leadership drifting around the campus, whether they were out in the parking lot or central supply or CAT scan or whatever, did not have direct communication with each other. So she secured more handsets, more mobile handsets so the leadership could talk amongst each other directly. The rest of us gained more supplies and prepared each room for whatever might come. It was both direct and indirect leadership. We got direction and then we self-assigned some of our own duty.

Hospital personnel streaming news on their office computers led to Internet system degradation. Ultimately clinicians depended on a combination of their own judgment and leadership messages to make patient care decisions.

So they were getting private phone calls and private texts from outside sources; who knows if they were actually on the scene? They were listening to their radio or texts from a friend. So all kinds of external stuff were coming but none of it was correct either. And then that was causing the nurses and staff down here to continue to follow whatever media station they could catch on their cell reception or the Wi-Fi reception we had here and that did not work well at all.

Our hospital system was overwhelmed. You couldn’t send pages. You couldn’t send emails. You couldn’t send out announcements because the system was completely clogged.

Security planning is often independent of medical care processes. As a result, communication between security and the hospital incident command system sometimes lagged. Respondents who worked with victim families noted there was some confusion about how to confirm family member identities and who was allowed to be with the patient.

Security had earpieces and nobody had access to what they’re hearing. They need to be sharing what they’re hearing. In the future I hope to get one administrator with the same equipment so administration isn’t closed off and we’re not left out of that critical information.

The use of social media sometimes added inaccurate information and unnecessary stress.

So that was kind of hard because we didn’t know what was real and was not real coming in.

Most clinicians felt prepared due to ongoing training drills.

ED training is both clinical-role specific, completed as a professional requirement, and interprofessional. Some respondents had completed active shooter training prior to the attack. Prior training had limited influence, however, since the attack was technically not an active shooter incident at their facilities.

Most participants said disaster drills were helpful because most people knew their role and task. Clinicians knew how to form response teams, prepare treatment beds/areas and assemble appropriate equipment. People moved to their roles without conflict. Clinicians felt confident and competent to treat patients regardless of condition. Two hospitals had recently held drills and respondents felt this was a contribution.

You know, I was just prepared for the worst. I didn’t know if there were shooters who were going to try and attack the hospital. Whatever it was, I was prepared for it. I was good, I’m going to protect my staff, I’m going to treat as many patients as I possibly can, we are going to do the best we can. I think everyone acted that way.

Respondents identified some training gaps, including working with non-clinical administrators and patient/family liaisons, increased people in the ED, and how to handle media. Some remarked that without media training they had to develop a response extemporaneously.

We had a lot of media turning into a circus. So they can video off the hospital front and we had patients coming in. A nurse mentioned, ‘why don’t we drop blankets and cover up patients to protect them from this media circus. These patients deserve privacy.’ We surrounded that patient as we brought them in and protected their identity.

Some expressed a desire to understand more about weapons and ballistics.

Especially when the detectives come to talk to you, you’re the only access they have to the patient and they’re asking you questions that you probably can’t answer because you’re not a ballistics specialist.

Lots of people want to help but they need direction to know where to be effective.

Everyone commented on the spontaneous help offered.

It made me very proud to work here – to be a part of it. To see how everybody wanted to help…to see that we were all here as a team…was amazing.

For the EDs, that meant doctors and nurses calling to offer clinical assistance, as well as local businesses dropping off food and water. When a bomb threat was called into one facility a local casino security force immediately brought bomb-sniffing dogs to the hospital. Those already working stayed significantly beyond their shifts. Unexpected help from qualified individuals already credentialed to work in the hospital was welcomed and represented an extended workforce. Calls came in from hospitals in adjacent counties offering operating space. There was a need for a better process for integrating (or not) volunteer clinicians into patient care in the ED.

It was crowded with the number of emergency physicians and trauma surgeons who were there and so pile on top of that people who came down wanting to help who really didn’t need to be there. That got in the way a little bit. But I think on our side, we did a pretty good job of policing that I mean nobody kicked anybody out, but there could have been a way I think to regulate better who was down there. It’s just human nature to want to help. It’s hard to be critical of that.

There could be something to the effect of a central station where all providers check in and are doled out to certain areas.

I think pairing off worked well when you have sets or surplus of staff where you can handle every patient that comes. You know, the way we paired off was one trauma attending, and one ED attending for each patient. I think that worked very well. And because we were so overstaffed accompanying those patients up to OR really worked for us.

The terrorist attack tested limits of responder security, media management, securely identifying patient families who should have access, and securing places where people might have access to view things they should not (i.e., rooftops of buildings). Help from outside agencies did provide needed support.

I mean we just felt completely safe. There were people everywhere. I knew their job was mainly to protect hospital staff but they weren’t just police officers in uniform, they had their vests on, their dogs and their guns, and their SWAT cars. It was a whole army of people…

Most responders were OK bypassing normal processes to expedite patient care.

All of the facilities activated some level of their disaster plan. MCIs may create scenarios where bypassing an approved process or policy is considered prudent to quickly treat more patients effectively. Most respondents reported some kind of process deviation and felt that decision was warranted by the circumstances.

I had 3–5 ambulances on delay on the wall; I wanted to consolidate patients and have one crew watching patients to release the other crews because I didn’t know how many patients were trying to get in. I was able to release one crew.

Multiple respondents documented moving patients out of the ED to admitted beds upstairs more quickly than the norm. In some cases this happened without written orders because of lack of access to computers, which is consistent with documented MCI responses.^4^ Additional deviation examples included patients taken to the operating room without computer physician orders, a blood bank moved to the ED, a low-acuity, non-trauma patient discharged without paperwork, briefly releasing emergency physicians to accompany patients transferring to the OR with surgeons, and transferring patients to ICU earlier than usual.

I did find one patient that was in severe respiratory distress. So I grabbed one of my emergency residents who was superfluous for the traumas and I said, “Do you want to intubate or do you want me to?” and he was very happy to intubate…then I called the I.C.U. attending and said, “I normally stabilize these patients down here for a while. Do you mind taking them up right now?” and it was delightful. They said, “Sure. Send them right up” even before I had a blood gas.

MCIs have personal and emotional impact on clinical responders.

Several respondents reflected on their personal situation while providing patient care, which was while the shooter identity was unknown and still at large.

I was scared for my family, honestly but not for anything that was happening in the ED or for my own safety. But my family’s I was.

Several had children in schools on lockdown. One facility received a bomb threat during this time. Clinicians did not know if they were treating victims or the shooter. Most respondents were not afraid, and expressed commitment to patient care regardless of their personal concerns.

Fear was not a factor in providing patient care. No one retreated, despite the threat of an at-large shooter, several physicians provided care in an open area established for disasters in the ED parking lot.

Most respondents said they employed their usual methods to deal with stressful days. Others felt that discussing their feelings in safe environments was key.

I was uncomfortable being out in large crowds after this. I did feel anxious coming into work. So it definitely impacted me personally. It hasn’t affected how I do things around here, professionally, because I think I can separate that. But definitely I do think it has impacted me personally.

I voiced a lot of concerns to my wife. The thing that may have helped … was realizing how much more other people have to process, like there was the big shoot-out where the police shot the shooters and I’m thinking, I’m coming home to a normal life after helping to save this patient, what about those police officers? They were just involved in a shoot-out, and killed somebody, but in the process probably saved, I don’t know, how many other lives?

Debriefing occurred in different settings post event and were typically held in conference rooms in the respective hospitals or attached campus grounds. Most respondents felt these were useful in processing the MCI. Many respondents commented that the event resulted in a lingering malaise that was difficult to shake for many weeks. Several people expressed gratitude for debriefing meetings that were mostly organized by clinical leadership and by clinical role (physicians separate from nurses). Two people commented they wished the debriefing had happened sooner and interprofessionally.

We talked about it amongst emergency physicians, trauma surgeons and nursing. I never really go home and think about patients ... I’m pretty good at brushing things off. Even though the actual patient care was no different than what we usually do, the context and knowing that it was this mass shooting and everything really sticks with you and obviously with all the news coverage and everything that occurs afterwards it was something that weighed on me for I would say at least a week.

While most felt that debriefing sessions were helpful in dealing with the incident, some felt that attending these were too painful and made them feel depressed and vulnerable.

My experience was debrief once and then do not talk about it. Forget it. Every time you talk about it, you’re going to have a nightmare, that’s what happened to me.

A few respondents felt that everyday security measures could be improved.

I think the best thing [hospital] has done is they’ve put in those metal detectors just like at the airports.

Many of the respondents felt a need to enhance their personal safety, mentioning being more aware of their surroundings, choosing when and where to walk, and considering training and/or acquisition of firearms.

I trained my family and my children to stay alert about surroundings…We teach them that we live in a different world. These things that are happening, it’s not pretty. It’s not what people should be doing to one another and bad things are happening and we need to be aware and protect ourselves.

### Practical Advice

All respondents offered practical advice for preparing for a MCI response. These are summarized in Table 4.

## DISCUSSION

Our qualitative study examined a MCI from the perspective of clinicians first caring for patients at area hospitals. Our study elucidated several themes to help other institutions prepare for similar incidents.

System resilience seemed bolstered by already established relationships. People relied on trust already developed from working together. They were proud about how a broad network of individuals, including prehospital responders, came together for a common goal. Literature documents that working together promotes resilience and training drills emphasizing “communicating, coordinating, and cooperating” promote social relationships because “emotional interaction may have a positive influence on team effectiveness.”^5^,^25^–^27^ Community relationships were also essential to obtaining water, food, and information. People worked at their level of training for the common goal and avoided power struggles because they already knew each other and what to do.

Social media was a two-sided issue. Consistent with similar events, responders used social media news reports to make decisions but not all information was accurate.^28^ Instead of relying on limited “disaster phones” distributed only to leadership, all respondents could group text or receive news updates. However, there was no designated authority to confirm information accuracy. Individuals made decisions based on a combination of their own judgment and leadership messages, but everyone yearned for timely, accurate updates. The healthcare system should “adopt, use and leverage social media,” but usage standards have not been established and are often at odds with the general public who are able to post pictures and information in real time that may violate privacy standards at care facilities.^28^

Most interviewees felt safe in the work environment and did not feel their fears impacted their work. EDs are high-stress environments and workers are often exposed to violent acts during their regular work.^29^ Their reported coping mechanisms were consistent with prior research about how protective skills and resilience develop after traumatic events. 30 Some described ways they were increasing their personal safety independent of work, including possible gun acquisition.^31^

Based on our interviews, debriefing sessions, in multiple contexts and venues, should be available, but not mandatory. Hospital disaster plans should include ongoing debriefing and counseling access, including individual follow-up with non-attenders. Sessions should address common maladies such as difficulty sleeping, increased fear and hypervigilance reported from other incidents.^16^ Lingering effects from MCIs may be exacerbated by personally knowing the victims and the randomness of events (e.g., it might happen again.), and women typically report more symptoms.^16^,^32^

## LIMITATIONS

Study limitations included clinicians who had the strongest negative impact from the incident may not have responded to our interview requests, which may have resulted in selection bias. Most of the interviews were from the Level I trauma center. Our sample under-represented nursing and administrative staff. The smaller sample size and time lapse between the event and interview completion may also hinder validity.

## CONCLUSION

This study provides context regarding the response of healthcare personnel from multiple institutions to a singular terrorist attack in the U.S. While most responders felt prepared, non-traditional communication channels, managing volunteer assistance, and corralling media presented novel challenges not included in current disaster plans. Developing post-event debriefing plans that acknowledge personal impact on providers should also be a priority. By understanding these common experiences, opportunities arise to prepare for future incidents. Additionally, knowledge gained from participants sharing their best practices allows both an occasion to review and improve individual current MCI plans as well as an opportunity to study methods described in future events.

## Figures and Tables

**Table 1:** Responder resources to the San Bernardino mass shooting.

Resource hospitals	Service area
Loma Linda University Emergency Department	Level I Trauma, Adult and Pediatric Patients
Arrowhead Regional Medical Center	Level II Trauma, San Bernardino County
Riverside University Health System Hospital	Level II Trauma, Riverside County
St. Bernardine’s Medical Center, San Antonio Regional Hospital, and Kaiser Permanente Fontana Medical Center	Community hospitals
Inland Counties Emergency Medical Agency (ICEMA)	Regional Disaster Response System. Oversees prehospital services in the area and provides opportunities for collaboration and integration.
REDDINET	Emergency medical communications network links hospitals, first responders, law enforcement, and public health assets.

**Table 2:** Standardized interview guide for mass shooting study.

Demographics	How long have you been at your medical center? How many years have you been in the emergency department? Where did you do your training? How old are you? What is your gender?
Grand Tour Question	Tell me about that day.
Overall Framing	What was your job title that day? (prompt: medical director, doctor on duty, nurse on duty, tech, etc.)
Process & Logistics	What worked well? What didn’t work well? Was there anything that didn’t work well? Was there anything you weren’t prepared for? Has anything changed in the emergency department as a result of these events?
Disaster Plan	Did you activate your disaster plan and if so, how did it go? Were you able to move low acuity patients out? Were you able to make room for more traumas? Did you call other hospitals? Have you had training to deal with active shooter events? If yes: Was this through your work or another venue? What aspects of the training you had were especially helpful? If no: Do you plan on attending training to prepare for this type of event?
Unexpected Outcomes	Did anything surprise you about the response? Some people we have spoken with at various sites have said they know certain hospital guidelines or rules were broken to care for patients that day. Are you aware of any hospital rules or guidelines that were broken that day?
Communication	How was the communication? Were there any disruptions or breakdowns? What would have improved communications? How was the electronic medical record?
Emotional State	Did you feel safe? Do you think that impacted patient care? How has your perspective regarding future threats changed? What impact has the terrorism had on you professionally? What impact has the terrorism had on you personally? (prompt: Did you do anything differently as a result such as changes to how you take care of yourself?) What would have helped you process the incident? Did you attend a debriefing? If yes: What aspects of the debriefing were helpful? Imagining future scenarios, would you play the same role or would you want to take on a different role? Would you respond the same way or a different way? What training or information do you feel you need to do in order to be better prepared next time?

**Table 3:** Demographic characteristics of interview subjects.

	Male (n=14)			Female (n=12)			Total (n=26)		

	Median	1st Quartile	3rd Quartile	Median	1st Quartile	3rd Quartile	Median	1st Quartile	3rd Quartile
Age	39.5	36.5	48.0	44.5	39.3	54.8	41.0	37.3	50.8
Years post degree	10.0	5.0	13.0	14.5	9.5	17.8	12.0	6.3	16.5

		n	%		n	%		n	%

MD/DO		12	46		6	23		18	69
Nurse or Admin		2	8		6	23		8	31

*MD*, Doctor of Medicine; *DO*, Doctor of Osteopathic Medicine; *admin*, administration.

**Table 4:** Practical advice for hospital response to mass casualty incidents.

Practical Advice	Create separate teams to care for patients already in the emergency department (ED) and the non-MCI patients who present to the ED.
	Create and distribute a paper list of disaster phone numbers of specific individuals/roles and designation of several individuals to serve as runners to communicate with people not immediately reachable by phone.
	Recognize the value of social media, especially peer-to-peer/text messaging, and plan for personal phone use instead of expecting people to only communicate via specific hospital disaster phones.
	Use of personal protective equipment, including gowns, can make it difficult to identify the roles of those providing patient care. Using stickers to identify individual roles as physician, nurse, or respiratory therapist, solves this issue.
	Integrating blood bank services into the disaster plan to temporarily bring blood supply into the ED while maintaining strict protocols.
	Encourage IT to plan for significantly increased streaming and Internet usage during MCIs. One hospital had to temporarily suspend Internet service because so many people were streaming news on their desktop computers it slowed the patient care activities that required IT resources.
	Out of concern for bombs or other weapons, decide in advance whether patients will have clothes removed prior to entering heavily populated trauma bays.
	Work with administrators to limit the number of extra people entering the ED by establishing a check-in system for volunteer clinicians. This plan needs to include a central place where people standing by also receive communication and updates about the disaster response and needs.
	Provide additional security to control who enters the ED, as well as identifying and controlling access to the hospital and grounds/parking lots surrounding the area.
	Immediately engage media in a single defined location to limit disruptive impact on patient care. Provide a direct liaison while remaining in control of where they can park to prevent blocking traffic flow of responders, patients and family.
	Establish a liaison to accompany family members of patients. The liaison may assist in obtaining identification, communication with providers, and to serve as a shield from media questions until the families are ready to manage it themselves.
	Develop a plan for debriefing of critical incidents that recognizes the personal and emotional impact on clinical responders.

*MCI*, mass casualty incident; *IT*, information technology.
